# Healthcare professionals’ experiences of providing care for patients with Graves’ disease: a qualitative interview study in Swedish secondary care

**DOI:** 10.1136/bmjopen-2026-119495

**Published:** 2026-10-06

**Authors:** Agneta Lindo, Sara Alsén, Andreas Fors, Helena Filipsson Nyström

**Affiliations:** 1 Department of Endocrinology, Sahlgrenska University Hospital, Gothenburg, Sweden; 2 Institute of Medicine, Sahlgrenska Academy, University of Gothenburg, Gothenburg, Sweden; 3 Wallenberg Centre for Molecular and Translational Medicine, University of Gothenburg, Gothenburg, Sweden; 4 Centre for Person-Centred Care (GPCC), Sahlgrenska Academy, University of Gothenburg, Gothenburg, Sweden; 5 Institute of Health and Care Sciences, Sahlgrenska Academy, University of Gothenburg, Gothenburg, Sweden; 6 Research, Education, Development and Innovation, Primary Health Care, Region Västra Götaland, Gothenburg, Sweden

**Keywords:** Thyroid disease, QUALITATIVE RESEARCH, Nursing research

## Abstract

**Abstract:**

**Objective:**

Graves’ disease (GD) is an autoimmune thyroid disease, mainly affecting women of working age. The hyperthyroidism in GD is characterised by physical symptoms (tachycardia, weight loss, sweating and tremors) as well as cognitive and psychological problems that have a major impact on the daily lives of patients. Mental symptoms are particularly distressing, with patients expressing a desire for more communication and support for the mental aspects of their care. The aim of this study was to explore the experience of healthcare professionals (HCPs) when providing care and treatment to patients with GD.

**Design:**

A qualitative study based on interviews conducted digitally or face to face was performed with data evaluated according to Graneheim and Lundman’s qualitative content analysis.

**Setting:**

The study was conducted within specialised care at the secondary level in a Swedish healthcare context.

**Participants:**

12 HCPs (6 physicians and 6 registered nurses) with experience in providing care and treatment for patients with GD were interviewed.

**Results:**

The study highlighted shortcomings in the care of patients with GD, emphasising that GD is more complex than many other endocrine conditions due to its cognitive and psychological impact. Several obstacles hindered the ability to meet patient needs, including limited time, an organisational structure that is not adapted to the nature of GD, insufficient resources and a lack of interdisciplinary cooperation. HCPs acknowledged that patients often had needs that exceeded what the current system allowed them to address, which led to frustration and affected the work environment. There was a strong and shared desire among HCPs for changes that could improve the quality of care for this patient group.

**Conclusion:**

Optimal care for patients with GD requires treatment tailored to the specific needs of each patient supported by organisational approaches that acknowledge the complexity of the condition, addressing both physical and psychological aspects. A way to achieve this is by enabling sufficient time for patient-HCP interaction, improving continuity of care and strengthening interdisciplinary collaboration. Key implications for practice include targeted training in the psychosocial aspects of GD and the integration of a contact registered nurse with specialised knowledge in thyroid diseases, which may improve both care quality and the HCP work environment.

**STRENGTH AND LIMITATIONS OF THE STUDY**

- The inclusion of healthcare professionals with varied clinical roles, experience in caring for and treating patients with Graves’ disease (GD), age and gender enhanced the diversity and richness of perspectives.
- Including both large and small clinics, they captured experiences across different organisational contexts and care structures.
- Working together, researchers with clinical experience and those with a more distant perspective contributed to the credibility of the interpretations.
- Patient and public involvement was integrated into the study.
- Recruitment from a limited number of clinics may reduce the transferability of the findings to other healthcare settings or geographical contexts.

## Introduction

 Graves’ disease (GD) (autoimmune hyperthyroidism) is a relatively common disease that primarily affects women in their working years.^1^ Symptoms are often distressing for patients with hyperthyroidism but tend to improve with treatment as hormone levels return to the normal reference range. A significant proportion of patients experience persistent mental symptoms and/or complications such as Graves’ orbitopathy (thyroid eye disease), which further hinders patients from recovering their quality of life.^2^ Worldwide, patients are managed either by general practitioners or by physicians specialised in endocrinology (endocrinologists) who provide antithyroid drugs (ATDs) in conjunction with the involvement of other specialists for surgery, radioactive iodine therapy and GO. In Sweden, care for hyperthyroidism often starts with the general practitioner who discovers abnormal tests, provides beta-blocking therapy and refers patients to endocrinologists for further management, including ATD therapy and decisions regarding long-term ATD treatment, thyroid surgery or radioactive iodine therapy.^3^

The estimated waiting time after referral for suspected GD is approximately 2 weeks; however, it is important to note that this timeframe may vary between hospitals, with some extending the waiting period for up to 4 weeks. Most patients only have contact with endocrinologists during scheduled visits and when receiving thyroid test results, and may contact counselling services, where nurses provide support as needed. Some clinics have registered nurses (RNs) who are more involved in the care process and conduct their own visits and follow-up with patients. However, this is not standard in all clinics, although team-based care is highlighted in the Swedish national guidelines as an important aspect of patient care.^3^

The national guidelines for hyperthyroidism in Sweden emphasise the importance of increasing patient involvement in their care. Introducing a contact RN with in-depth knowledge of thyroid diseases can be a way to increase safety and, at the same time, strengthen the patient’s ability to be actively involved in their own care.^4^ Nexø and colleagues^5^ have highlighted the key role of healthcare professionals (HCPs) in caring for patients with hyperthyroidism. Two studies have explored patient experiences of GD, with one addressing the later stages^5^ and the other the early phase.^6^ Both studies found that the psychological and cognitive impact of the condition was central to patients’ experiences, yet these aspects were not consistently addressed by HCPs.^5 6^ The findings suggest a need for a more consistent point of contact to which patients can turn for questions and support.^6^

Existing research on the care and organisational management of GD is limited and there is a lack of evidence-based guidance on the optimal working approach for this patient group. As the disease often presents with mental symptoms such as fatigue and difficulties with concentration and memory in the hyperthyroid state, it is crucial to explore the perspectives of HCPs on their relationship with patients to promote favourable outcomes, ensure patient safety and enhance quality of care. Engagement of HCPs has also been shown to correlate with patient safety.^7^ Healthcare provision should aim to enhance patient care experiences, improve the overall health of the population and reduce per capita healthcare costs.^8^ The achievement of these three objectives depends on an effective healthcare organisation and an engaged healthcare workforce.^8^ The aim of this study was to explore the experiences of HCPs in providing care and treatment for patients with GD.

## Methods

### Study design

This qualitative interview study involved physicians and RNs working in secondary referral centres for patients with GD, where they provide care and treatment. A qualitative design was chosen to obtain an in-depth understanding of HCPs’ experiences, needs and emotional responses related to the care of patients with GD. A semi-structured interview guide was developed to allow participants to describe their experiences and perspectives while ensuring that relevant areas were addressed across interviews. The interview data were analysed using qualitative content analysis as described by Graneheim and Lundman and further developed by Graneheim *et al* and Lindgren *et al.*^9–11^

### Participants and setting

Participants were recruited from endocrinology outpatient clinics across Sweden, with the majority working in the Västra Götaland region. To ensure a broad perspective, we included both physicians and RNs from three smaller hospitals and two regional hospitals, representing a range of clinic sizes. To enhance variation in experiences and perspectives, participants were recruited from different professional groups and from hospitals of varying sizes and geographical settings. Larger hospitals have a high influx of patients with GD, who interact with physicians through regular visits, digital consultations, telephone calls or written correspondence. RNs in these settings typically encounter patients during intravenous methylprednisolone infusions for GO or via phone when patients have questions. Smaller clinics treat fewer patients but use a care structure similar to larger hospitals, involving both physicians and nurses.

Study information was provided at endocrine outpatient clinics through verbal communication and informational flyers. The flyers allowed HCPs to register their interest in participating and to obtain further information from the research team. Two RNs, including the second author, who had no prior relationship with the participants, were responsible for contacting, scheduling and interviewing the HCPs who consented to participate. To be eligible, participants had to be employed as an RN or physician in an endocrine outpatient clinic and have experience providing care and treatment to at least 20 patients with GD within the previous 3 years to ensure that participants had sufficient recent experience of providing care and treatment to patients with GD. Participants were given the option to conduct the interview digitally or face-to-face.

### Data collection

A total of 14 individuals were invited to participate, of whom 12 consented and were interviewed. Interviews were conducted between January 2023 and October 2023. The interviews lasted on average of 43 min (range: 20–90 min).

These interviews took place in a setting where the participants felt comfortable, with ten conducted digitally and two conducted face-to-face. All interviews were audio-recorded and transcribed verbatim by the two RNs who performed the interviews. The aim of the study was explained to the participants and examples of key questions from the interview guide included: ‘How do you experience meeting, caring for and treating patients with GD?’, ‘What care and support do you believe these patients need?’ and ‘How do you perceive that the care interventions are adapted to patient needs?’. Follow-up questions such as ‘Could you elaborate on that?’ or ‘Can you provide an example?’ were asked throughout the interview to gather detailed responses. The interviews continued until the research question was deemed fully addressed. The characteristics of the participants are shown in table 1.

**Table 1:** Characteristics of the participants

	No. (%)
Sex	
Female	9 (75.0)
Male	3 (25.0)
Age (years)	
31–40	2 (16.7)
41–50	6 (50.0)
51–65	4 (33.3)
Profession	
Physician	6 (50.0)
Registered nurse	6 (50.0)
Experience in endocrinology care and treatment (years)	
<5	2 (16.7)
5–10	3 (25.0)
>10	7 (58.3)

### Data analysis

Qualitative content analysis was applied.^9–11^ AL and SA were primarily responsible for the analyses, which were conducted in close collaboration with the co-authors (AF and HFN). Initially, there was an open reading of the data to obtain a general perspective in relation to the aim of the study. Meaning units representing HCP experiences in providing care and treatment for patients with GD were identified, condensed and coded.

The analysis initially focused on the manifest content of the interviews, ie, the visible and directly expressed aspects of the participants’ experiences. Meaning units were identified and coded, and these codes were analysed and compared for similarities and differences. Related codes were grouped into sub-categories, which in turn formed distinct categories reflecting broader patterns in the data. The process of condensation aimed to preserve the core meaning of the text, capturing essential information and details regarding diverse care experiences related to GD.

Through a process of reflection and discussion, the analysis moved beyond the manifest content to interpret the latent content, the underlying meanings embedded in the participants’ narratives. This interpretive phase aimed to capture the more implicit, often unspoken, aspects of HCP experiences of caring for and treating patients with GD. Sub-categories and categories were revisited and re-examined with attention to patterns, tensions and deeper meanings, ultimately leading to the formulation of an overarching theme that encapsulated the shared essence of their experiences.

The research team brought diverse perspectives to the analysis. AL contributed with research and occasional clinical experience with GD, while HFN had extensive clinical and research expertise within the field. In contrast, SA and AF lacked direct experience of patients with GD, which added a layer of critical distance.

The team continuously reflected on their preconceptions derived from previous clinical and research experiences related to GD, fatigue, exhaustion disorders and qualitative methodology. Alternative interpretations were actively explored and discussed within the research team, supporting a final interpretation firmly grounded in the data and providing a coherent understanding of the HCPs’ perspective.

Trustworthiness was considered throughout the research process in terms of credibility, dependability and transferability.^9–11^ Credibility was supported by including HCPs from different professional backgrounds and clinical settings and by using follow-up questions to obtain rich descriptions of their experiences. Dependability was supported through continuous discussions within the research team regarding the analytical process and interpretation of data. The participants, clinical context, data collection and analytical process are described in detail to enable readers to assess the transferability of the findings to other contexts.

## Results

The results highlight the experience of HCPs through the overarching theme ‘Inadequacies in care for patients with GD’ structured into three categories and seven subcategories (figure 1).

**Figure 1:**
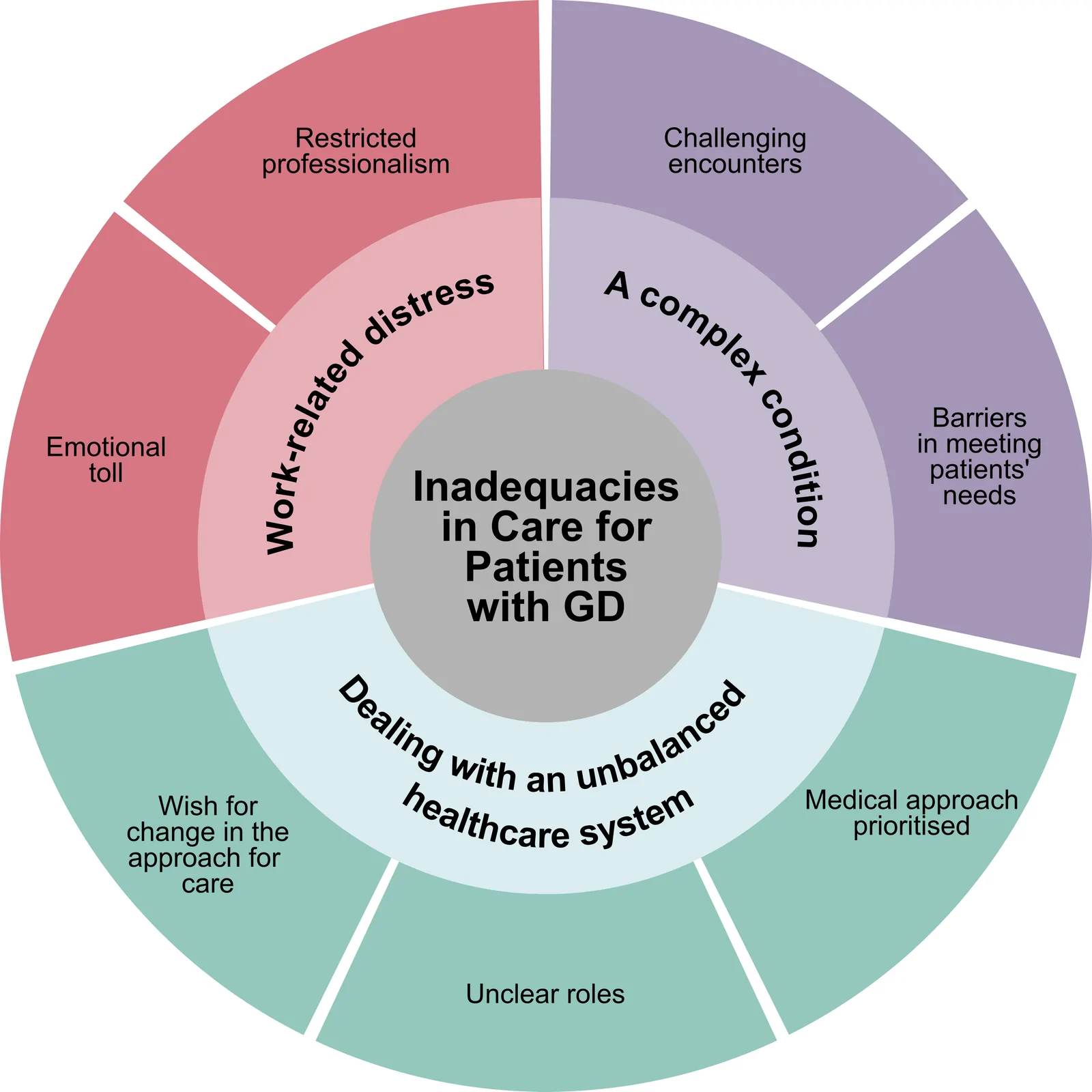
An overview of the overarching theme, categories and subcategories from the interviews with healthcare professionals providing care and treatment for patients with Graves’ disease (GD).

### Inadequacies in care for patients with GD

The overarching theme highlights that providing care and treatment for patients with GD was perceived as more complex than for patients with other endocrine conditions due to the severity of symptoms combined with limited organisational structures and a lack of care intervention tailored to patient needs. The HCPs described experiencing work-related stress and viewed the healthcare organisation as insufficient to meet the needs of patients with GD. They expressed a wish for more comprehensive support beyond medical treatment and for improved interdisciplinary teamwork, both of which were not part of standard practice in the clinics.

### A complex condition

#### Challenging encounters

HCPs described that patients with GD revealed distressing mental symptoms such as stress, frustration, anxiety and impaired cognitive function that complicated communication and the establishment of mutual understanding. The stress and confusion expressed by these patients required HCPs to adopt a more complex care approach, demanding more effort compared with conditions where patients’ mental states were less of a concern. “I notice a significant difference in how I approach patients with Graves’ disease. With patients who have other endocrine conditions, I can be more flexible, joke around and have a more relaxed tone. However, I need to be calmer and more cautious with these patients to avoid contributing to their stress or anxiety*.”* (#5, physician).

HCPs needed more guidance and support to ensure that patients fully understood and were able to process medical instructions and treatment plans. One RN stated, *“*They stand out more, these patients need more advice and support, and therefore perhaps a more open way than they might have today compared with some other patient groups.*”* (#2, RN). This added complexity required a more tailored and responsive approach, as miscommunication could worsen patients’ distress or hinder their engagement in treatment. HCPs also experienced a duality in providing care and treatment for patients with GD, finding it both challenging and intellectually stimulating. The multifaceted nature of the condition, with its broad range of symptoms and tailored care demands required deeper clinical insight and flexibility in treatment strategies. However, the long-term nature of managing GD allows HCPs to build lasting relationships with their patients, offering both personal and professional fulfilment as they witnessed gradual progress and contributed to the patients’ overall well-being over time. *“*Yes, but I think it’s an interesting and exciting patient group with many different challenges… They have various symptoms that need to be considered. And then it’s also long-term care that you must work with. You end up having prolonged contact with these patients.” (#10, physician).

#### Barriers in meeting patients’ needs

HCPs expressed that patients with GD had needs that exceeded the HCPs’ available resources and the current healthcare organisation could adequately address. The organisation of care for patients with GD was characterised by an almost exclusive focus on physician visits aimed at establishing a diagnosis and adjusting medication. As physicians had fully booked schedules, there was rarely room for addressing the patients’ broader needs.

HCPs experienced that the patients were often stressed and sometimes limited in their ability to follow recommendations due to the standard time for a patient encounter being too short to meet their needs. “I think on most occasions I am stressed because I know there is a time limit and this is very challenging.” (#4, physician). RNs often faced structural and competency-related barriers that prevented them from fully addressing the needs of patients. A key obstacle was the lack of both specialised knowledge and a formal mandate within the clinic to provide structured care for patients with GD. In contrast, some RNs felt confident in their knowledge and experience, while others struggled with uncertainty about their ability to provide adequate care. One RN expressed, “Unlike mastering diabetes on your five fingers, I feel very inadequate here because I don’t really have the knowledge to answer their questions*.”* (#3, RN).

### Dealing with an unbalanced healthcare system

#### Medical approach prioritised

Care was primarily structured around medical procedures and standardised treatment protocols, such as ATD adjustments and clinic visits, rather than promoting patient-provider relationships or offering long-term support. The planning and delivery of care and treatment were predominantly guided by the medical diagnosis and its symptomatology, focusing on measurable biological markers and anticipated disease progression. The medical perspective, with almost exclusively physician-led visits, could pose potential risks as patients often met with different physicians who lacked knowledge of the patients’ individual needs. Moreover, due to the varied roles of endocrinologists within the clinic, patients often encountered multiple physicians, which not only fragmented their care experience but also contributed to inconsistencies in communication and treatment recommendations. Despite all physicians being well acquainted with local routines, the involvement of different physicians sometimes resulted in increased variation with respect to medical interventions. “There is a very rotating group of physicians, which also means that it can sometimes be a bit frustrating as different physicians can give different messages or information*.”* (#2, RN). This medically oriented approach sometimes overshadowed broader perspectives on patient health. Aspects such as the psychological burden of illness, social disruptions or how daily life was affected by symptoms were often given less attention or excluded entirely from routine follow-up.

At smaller clinics, there was greater accessibility to contact RNs who could schedule appointments to provide patients with information, education and support according to the individual needs of the patients. These clinics could also offer contact with a smaller number of HCPs, which promoted tailored care and a sense of security for patients. The continuity of care and familiarity with the same HCPs were described as beneficial, contributing to increased trust and a more holistic support. One physician mentioned, “Here, we have good accessibility and they have the same healthcare professional or contact throughout their care period, mainly through physician visits.*”* (#10, physician).

#### Unclear roles

RNs were a constant presence in all endocrinology clinics, but their roles and responsibilities varied. Within the organisation, RNs did not have a formally defined caregiving responsibility for patients with GD, nor was such a responsibility explicitly required by managers. This differed from their structured role in diabetes care, where RNs are responsible for most care interventions and physicians have a more consultative role. For patients with GD, RNs primarily conducted blood tests and administered medical treatments but their role in ongoing patient support was undefined, limiting their interactions to occasional phone contact or assistance during medical examinations. RNs were already overburdened and the unclear role expectations made it difficult to establish a consistent approach to patient care. It was unclear why patients with GD were not linked to caregiving efforts in the same way as patients with other conditions (eg, patients with diabetes). One RN remarked, *“…*and the idea is also to refer it to the physicians because we barely have time to take care of our patients with diabetes as it is.*”* (#3, RN). Several RNs indicated that their involvement in GD care was not formally structured, leading to inconsistencies in the care provided to patients. Without an established framework, the extent of an RN’s caregiving role depended on individual interpretation rather than standardised guidelines. This role of uncertainty was also evident among physicians, who expressed uncertainty about what responsibilities RNs should have in GD care.

Many of the patients’ queries focused on diet, exercise, sick leave, workplace issues and cognitive impact. Despite the presence of RNs, the lack of a defined interdisciplinary team created gaps in support, though a more cohesive team was seen as beneficial. One physician highlighted the need for clearer role distribution. “Yes, and with teamwork, not because it doesn’t exist. There is, how can you say, the spirit of teamwork exists but there are important functions missing. A physician cannot fulfil all functions. A physician cannot be a psychologist, an RN, an occupational therapist or a counsellor*.”* (#8, physician).

#### Wish for change in the approach for care

Many participants described that inadequate care for patients with GD could be addressed by appointing a contact RN who would take greater responsibility throughout the care trajectory. A recurring suggestion was the need for RNs to specialise in thyroid diseases, as with diabetes care, and take on a clearly defined role in coordinating care and providing patient support. This would allow for a more nurse-involved model, fostering both continuity and accessibility. One physician reflected that, *“*I would have liked to have a nurse who specialises in this area*.”* (#7, physician), highlighting the desire for more tailored nursing expertise.

Participants felt that such a shift could ease the pressure on physicians, while simultaneously improving the care experience for patients. For instance, one RN described how implementing a nurse-led model could create development in the profession and provide career opportunities, “…have more of a nurse-led approach, as is the case in some other hospitals. That seems fun to me. For me as a nurse, as career development, as development in the profession*.”* (#12, RN).

Alongside the wish for a contact RN, participants emphasised the need for interdisciplinary collaboration. The lack of a clear care team made it difficult to address patients’ broader needs. HCPs called for a broader care team, including both psychologists to address significant mental health issues, social workers to assist with financial and social challenges and occupational therapists to support return-to-work processes. These professional roles were not part of the care team. One physician said, “Compared with other endocrine diseases, I think this group requires much more support, multidisciplinary support*.”* (#7, physician). Physicians expressed frustration over the absence of referral options to other professionals, such as psychologists, social workers or occupational therapists. They recognised the importance of someone with insight into the entire care process, someone the patient could turn to for both medical and psychosocial support. A more structured team-based model of person-centred care (PCC) was therefore proposed, in which different professions contribute according to each patient‘s needs and resources. RNs described the need for more knowledge and resources to meet these needs, stating that, *“*Graves’ disease is very extensive and broad, so you need a lot of knowledge and education*.”* (#1, RN). In this model, the RN would assume a central caregiving role, providing continuity, availability, follow-up and tailored interventions in collaboration with other professions.

### Work-related distress

#### Emotional toll

Participants described the complexities of managing patients with GD, particularly those who expressed a pressing need for symptom relief and stability. These interactions were not only clinically demanding but also emotionally challenging, requiring constant effort to reassure and support patients who often felt frustrated with their ongoing symptoms. The HCPs noted that a workday filled with multiple appointments for patients with GD could feel overwhelming, especially in certain units where entire days were dedicated solely to patients with GD. As one RN expressed, “And yes, sometimes it can be frustrating because you don’t have unlimited time and you see how it just ticks on with more and more calls and errands and so on. And then, you’ve been sitting on the phone with someone for half an hour, you don’t really have time for that*.*” (#2, RN).

HCPs frequently encountered a sense of frustration from patients who, despite consistent treatment efforts, continued to struggle with distressing symptoms. As one physician expressed, *“*You give treatment but then the patient doesn’t always feel well again and you don’t really know how to help the patient” (#7, physician).

This ongoing challenge left many participants feeling inadequate, as they often found themselves unable to fully relieve the distress experienced by patients. Despite their efforts to adjust hormone levels and provide reassurance, the unpredictable progression of GD symptoms made it difficult to offer patients the relief they wanted. Although they worked thoroughly to balance and stabilise patient hormone levels, the complexity and variability of GD symptoms meant that patients frequently reported persistent discomfort or anxiety. For many HCPs, this sense of professional helplessness contributed to their emotional exhaustion, reinforcing feelings of frustration and fatigue. The cognitive load of explaining complex treatment plans while addressing patient concerns added to the daily mental burden. They emphasised the importance of guiding each patient through every aspect of care thoughtfully and comprehensively rather than rushing through explanations. However, this careful approach, while necessary, left them feeling mentally drained by the end of the day. One physician described, *“*I feel that it is mentally demanding.” (#9, physician). The emotional and cognitive toll of balancing patient needs with the responsibility of delivering quality care often left them with little mental or emotional energy remaining after a full shift.

#### Restricted professionalism

HCPs described experiencing a sense of restricted professionalism, where they felt unable to fully exercise their roles and responsibilities in line with their competence, values and professional ethics. Despite their willingness and ability to provide high-quality care, they expressed being constrained by organisational structures and time pressures. This led to frustration, as they were prevented from practising in a way they believed was in the best interest of the patient, leaving them feeling that their professional skills and intentions were not fully utilised in daily clinical work. One RN expressed an inability to provide essential support for patients’ well-being, *“*It’s a rather complex patient group, and these patients are very unwell and need quite a lot of support. I feel that this support is not always available and there isn’t always the opportunity or the time for us nurses*.”* (#2, RN). Many described how stress and time shortage restricted their professional judgement, reduced opportunities for reflection and, ultimately, affected their ability to deliver high-quality care. One physician reflected that, *“…*a stressed physician reflects their stress to the patient.” (#8, physician), illustrating how emotional strain directly influenced the care encounter. The inability to offer the kind of attentive, tailored care they believed patients with GD required was a recurring concern, contributing to feelings of dissatisfaction and powerlessness in their roles.

Several participants pointed out that the current work environment left little space for ongoing learning or interprofessional collaboration, which further limited their professional development. The high workload created not only physical exhaustion but also cognitive and emotional fatigue, affecting decision-making and the quality of care provided. One physician shared, *“*Sometimes the workload is so high that you are so stressed and it creates a fear of making mistakes. I think work conditions should be sustainable*.”* (#7, physician). This feeling underscored the tension between professional responsibility and the organisational limitations that hindered their ability to meet patient needs safely and effectively.

## Discussion

The overall underlying result from our study is the perceived inadequacy in providing care and treatment for patients with GD, which is influenced by challenges related to patient interactions, organisational structure and professional capacity. This sense of inadequacy led HCPs to feel unable to provide the necessary care and treatment, which could affect both the work environment, patient safety and patient outcomes.

Our findings suggest that these shortcomings may pose a threat not only to the well-being of HCPs but also to the quality of care. Previous research has shown that physicians and RNs who are continuously exposed to high demands, limited resources and heavy workloads are at risk of burnout when they feel unable to meet expectations.^12–15^ Moreover, the well-being of HCPs has been identified as a key indicator of healthcare quality^16 17^ and higher employee engagement has been linked to improved patient safety.^7^ Employee engagement is shaped by factors such as available resources, organisational culture, financial stability, leadership support and professional development opportunities.^18^

Our study highlights a lack of resources and an organisational culture misaligned with the needs of both HCPs and patients, originating from the nature of the disease. This can result in frustration and a sense of inadequacy among HCPs, particularly when they are unable to deliver the level of care required to ensure patient safety and well-being. Feelings of inadequacy are associated with an increased risk of mental health problems, such as depression and anxiety, which in turn impact both staff well-being and the quality of patient care.^12 13 19^ Furthermore, research indicates that these feelings may be linked to poor decision-making and a reduced willingness to cooperate. In some cases, HCPs may withdraw from patient contact as a means of protecting themselves from stress and pressure.^12 19^ A previous review has highlighted that factors such as psychological strain, workload and a non-supportive team climate may impair team performance, increasing the risk of miscommunication, treatment errors and compromised patient safety.^20^ Mambrey *et al*^21^ found that psychosocial working conditions, including high workload and lack of social support, were associated with an increased risk of slips and lapses, as well as challenges in patient interactions, further exacerbating the risk of compromised care quality.

Previous research has also shown that patients often feel disregarded when HCPs focus primarily on laboratory results rather than overall patient well-being.^5^ This aligns with our findings, which indicate that medical approaches were prioritised over holistic care. In our study, most clinical appointments were focused on physicians initiating and evaluating medication regimens. Physicians operated within a strained healthcare system, often working without a team to support them. RNs reported limited knowledge of GD and described their practice as largely confined to medical tasks, such as blood sampling and test result management, rather than encompassing the broader nursing responsibility mandated by professional, legal and ethical frameworks.^22–24^ According to PCC principles, it is essential to consider all aspects of a patient’s experience, not just clinical markers, to minimise the risk of overlooking crucial information relevant to their treatment and recovery.^25^

Implementing a team-based approach in healthcare has been shown to foster a more supportive work environment for HCPs while also enhancing patient care experiences.^26^ Patients particularly benefit from improved communication, continuity and clarity when strong collaboration exists between physicians and RNs.^20 26^ Effective teamwork has been linked to increased patient safety and higher-quality care.^27–29^ However, our findings reveal a lack of teamwork, despite HCPs expressing a strong desire to improve collaboration. Shanafelt and Noseworthy^19^ noted that limited resources present a significant challenge, yet research highlights that effective leadership, which fosters a culture of shared decision-making, is a key factor in developing high-performing teams. Establishing such teams can help reduce errors, boost employee engagement and strengthen team cohesion, ultimately leading to a safer and more fulfilling work environment.

Similar multidisciplinary and nurse-led care models have been implemented in other chronic diseases. In rheumatoid arthritis, nurse-led care has shown clinical and patient-reported outcomes comparable to physician-led care, with benefits for some outcomes, including patient satisfaction.^30^ In diabetes care, specialist nursing services have been associated with improvements in aspects of clinical care and reduced length of hospital stay.^31^ Although these findings cannot be directly transferred to GD, they illustrate how specialist nursing roles may contribute to the management of chronic and complex conditions. In relation to our findings, a more clearly defined role for specialist nurses in GD care may therefore be relevant for addressing some of the organisational and supportive challenges identified in the present study, including the need for greater continuity, patient support and coordination of care.

The HCPs in our study perceived that the needs of patients with GD were not being fully met and recognised their own inability to provide necessary care. The current healthcare structure does not adapt its organisation to the specific characteristics of different conditions, patient symptoms or their circumstances, which hinders the provision of PCC. This finding is supported by previous studies demonstrating that organisational structure significantly impacts healthcare quality.^32^ Patient interviews confirm this issue, revealing a lack of opportunity to discuss the psychological and cognitive effects of GD and a need to feel understood and validated.^5 6^ Many patients expressed that they no longer recognised themselves, their personalities, behaviours and well-being, which aligns with the experiences reported by HCPs. A structural change is needed to improve patient quality of life and sense of security while also enhancing the work environment and the ability of HCPs to provide high-quality care.

To the best of our knowledge, this is the first interview study to explore HCP experiences of providing care and treatment for patients with GD. Healthcare in Sweden is tax-funded and, although taxes are high, resources remain limited. The organisation of healthcare differs across countries and a European study has shown that working environments vary depending on the gross national income per capita.^33^ In countries with higher gross national income per capita, specialists tend to have lower patient volumes, fewer work in private practice and gender distribution differs, indicating that national economic factors influence working conditions.^33^ Therefore, the findings from the present study may also be relevant to HCPs in countries with similar organisational and economic conditions.

A more comprehensive team-based model has also been advocated in the national Swedish guideline for hyperthyroidism to bridge the gap recounted by patients.^3 34^ Our findings support a more clearly defined role for specialist nurses in GD care. A recent Swedish cost–utility analysis suggested that more cost-effective treatment strategies within the national guideline may enable the introduction of a specialist thyroid nurse as part of a team-based model to GD care.^35^ However, a shortcoming was the lack of education in thyroid diseases for RNs and, to overcome that barrier, the regional process team for hyperthyroidism in the Västra Götaland Region of Sweden has implemented education equivalent to 7.5 credits at the University of Gothenburg.^36^ This study highlights the need for organisational changes in the care of patients with GD. In a tax-funded healthcare system, such changes also require engagement at the policy level to increase awareness of the needs of patients with thyroid disease and to support sustainable improvements in care delivery. After years of lobbying, the patient organisation has impacted the government to include patients with thyroid disease in the special initiative to highlight female health.^37^

### Strengths and limitations

A strength of the study is the diverse participant group in terms of experience of working with GD, gender, profession and age, providing a well-rounded perspective.

The interviews were conducted at two large and several smaller clinics, which should be considered when assessing the transferability of the findings to other clinical settings. While responses from the smaller clinics aligned with those from the larger clinic, additional interviews at larger clinics could have further enriched the data.

Furthermore, this study was conducted within the Swedish publicly funded healthcare system, which should be considered when assessing the transferability of the findings. Although several of the challenges identified, including time constraints, fragmented care pathways and insufficient psychosocial support, have also been described internationally, differences in healthcare organisation, professional roles, access to specialist care and resource availability may influence the applicability of the findings to other healthcare settings.

To minimise the risk of bias, it is important to note that, although AL and HFN had prior knowledge of and professional contact with some of the participants, AL and HFN did not conduct the interviews or listen to the recordings. Instead, AL and HFN worked with de-identified transcripts during the analytical process, which contributed to a more objective analysis of the data. The analysis was primarily conducted by AL and SA with input from the other co-authors throughout the analytic process. A potential limitation is that, to preserve participant confidentiality, AL relied solely on the transcribed material and did not have access to the audio recordings. However, the combination of clinically grounded insider perspectives and analytically distanced outsider perspectives strengthened the credibility and analytical depth of the interpretation.

## Conclusion

Our study indicates that HCPs perceive a need for organisational changes to better address the needs of both patients and HCPs given the complexity of GD and its psychological and cognitive impact. Perceived organisational inadequacies among HCPs may increase the risk of burnout and pose a threat to quality of care and patient safety. To support optimal care, greater continuity, sufficient time for patient-HCP interaction, targeted training in psychosocial aspects of GD and strengthened teamwork are required. The integration of a contact RN with specialised knowledge in thyroid diseases may enhance continuity of care and collaboration within the healthcare team, thereby contributing to improved patient outcomes and a more sustainable work environment for HCPs. Future intervention studies should evaluate whether multidisciplinary PCC models, specialist thyroid nurses and structured psychosocial support can improve patient-reported outcomes and continuity of care in GD. The findings are relevant to HCPs involved in GD care, as well as to clinical managers and policymakers, by highlighting the link between organisational conditions and HCPs’ ability to provide comprehensive, PCC that addresses both disease-related outcomes and patients’ broader needs.

## Data Availability

No data are available.
