# Supplementary material for: Healthcare professionals’ experiences of providing care for patients with Graves’ disease: a qualitative interview study in Swedish secondary care
Source: BMJ Open. 2026 Oct 6;16(10):e119495. doi: 10.1136/bmjopen-2026-119495 (PMC13647001; doi:10.1136/bmjopen-2026-119495)
Supplement: Supplementary file 1 [file bmjopen-2026-119495.reviewer_comments.pdf]

PEER REVIEW HISTORY

BMJ Open publishes all reviews undertaken for accepted manuscripts. Reviewers are asked to complete a checklist review form and are provided with free text boxes to elaborate on their assessment. These free text comments are reproduced below.

ARTICLE DETAILS

Title (Provisional)

Healthcare professionals' experiences of providing care for patients with Graves' disease: a qualitative interview study in Swedish secondary care

Authors

Lindo, Agneta; Alsén, Sara; Fors, Andreas; Filipsson Nyström, Helena

---

VERSION 1 - REVIEW

**Reviewer**	**1**
**Name**	**Horie, Ichiro**
**Affiliation**	**Nagasaki University, Endocrinology and Metabolism**
**Date**	**29-Apr-2026**
**COI**	**None**

Authors conducted an interview-based study with healthcare professionals to investigate if patients with Graves disease have any inadequate conditions and its details.

However, the participants' number was very small, and the findings obtained from the study were difficult to be generalized scientifically. The article is not acceptable for publication in the journal as an original article.

**Reviewer**	**2**
**Name**	**Ganie, Mohd Ashraf**
**Affiliation**	**Sher-i-Kashmir Institute of Medical Sciences, Endocrinology**
**Date**	**11-Jul-2026**
**COI**	**None**

ORIGINALITY

This manuscript addresses a significant but often neglected angle in thyroid care. Most Graves' disease research focuses heavily on drug efficacy, radioiodine, or surgery. While

patient-reported quality of life has gained more attention recently , this paper takes a step back to look at the care environment itself through the eyes of the clinicians.

Investigating how both doctors and nurses experience care delivery in secondary referral centers is a fresh and necessary perspective. It moves beyond simple "resource shortages" to show how rigid clinical structures can actively restrict professional agency. This is a solid, original contribution to health services research that expands nicely on the authors' early pilot work in this area.

Importance of work to readers

The findings are relevant to anyone working in public healthcare, especially in tax-funded systems where resources are constrained. By showing that normal blood tests do not equate to a fully restored patient, the paper challenges clinicians to rethink how they manage Graves' disease. It is particularly useful for clinic managers and policymakers because it directly links scheduling pressures and a lack of role clarity to staff distress and potential risk of errors.

To understand how this study impacts different stakeholders, the key practical takeaways can be summarized as follows:

1. Researchers: The study reveals a critical gap in health services research, showing that clinical recovery (normalizing lab results) does not automatically mean psychosocial recovery. This highlights a need for future mixed-methods trials focused on integrated, person-centered care models.

2. Clinicians: It validates the daily, often unaddressed frustration of managing complex cognitive deficits within short, standard appointment slots. It highlights the value of shifting toward interprofessional, nurse-supported models.

3. Patients: It explains why their cognitive and emotional struggles are frequently missed during brief medical visits and provides evidence that patient advocacy groups can use to lobby for structured, dedicated pathways.

4. Educators: The paper exposes a gap in training regarding the neuropsychiatric aspects of autoimmune thyroid diseases, showing a clear need to integrate communication and psychosocial care into medical and nursing curricula.

5. Policymakers: It directly connects rigid clinic scheduling to clinician distress and potential safety risks, providing concrete data to support funding for dedicated thyroid contact nurses.

Evidence-based medicine

The qualitative methodology is straightforward and reliable. The authors used Graneheim and Lundman's method of qualitative content analysis, which is standard in nursing and health services research. They describe their analytical steps clearly—how they went from raw interviews to meaning units, codes, categories, and finally a latent theme.Using investigator triangulation—mixing clinical insiders (endocrinologists) with analytical

outsiders (nurses with no prior connection to these clinics)—helps keep bias in check. While the data is Sweden-specific, the structural issues they describe (brief visits, rigid funding, and a focus on biochemistry) are common in public specialist care globally, making these findings highly transferable.

Research question

The research question is clear: to explore the experiences of healthcare professionals when providing care and treatment to patients with Graves' disease. The paper answers this well. Instead of just listing what is missing in the clinics, the authors analyze the tension between the patient's neuropsychiatric reality (brain fatigue, anxiety) and the clinical environment's limits. They show how this clash leads to moral injury and clinical frustration.

Study design

An exploratory qualitative design using semi-structured interviews is the right choice for this objective. Interviewing both physicians and registered nurses gives a complete view of the care pathway. A sample of 12 participants is small but typical for this kind of qualitative study, especially since the interviews were long (averaging 43 minutes, up to 90 minutes) and provided rich, dense data that clearly reached thematic saturation.

Participants

The participants are well-described, and the corresponding patient profiles they manage are clearly characterized

1. Interprofessional Sample: The study consists of 12 healthcare professionals, split evenly between 6 physicians and 6 registered nurses, ensuring a balanced view.

2. Gender and Age: The sample is 75% female and 25% male, representing a realistic demographic spread in endocrine healthcare settings, with the majority of participants aged between 41 and 50 years.

3. Clinical Experience: The cohort is highly experienced, with 58% having over 10 years in endocrine care, and 25% having 5 to 10 years of experience.

4. Inclusion Criteria: Participants had to have managed at least 20 Graves' disease patients in the last 3 years to ensure their insights are active and current.

5. Target Patient Profile: The clinicians primarily manage working-age women (reflecting the local Swedish incidence of 21 per 100,000 annually) who present with physical symptoms combined with severe neuropsychiatric and cognitive issues like brain fatigue, anxiety, and memory loss.

Methods

The methods are clearly described. Recruitment spanned two large regional hospitals and three smaller specialist clinics in Sweden, giving a good mix of organizational settings. The interviews were conducted by two nurses who had no prior relationship to the participants,

which helps reduce social desirability bias.Regarding ethics, the authors consulted the Swedish Ethics Authority. Since they did not process sensitive patient data, formal approval was not legally required under Swedish law, but they still followed standard ethical practices, obtaining informed consent and maintaining confidentiality.

Results

The results are laid out logically under three primary categories:

1. A Complex Condition: This category incorporates challenging encounters and barriers to meeting needs. The findings show that neuropsychiatric symptoms make communication difficult, and standard short visits are simply insufficient for patients who struggle to process complex medical information.

2. Dealing with an Unbalanced Healthcare System: This focuses on how the medical approach is prioritized and roles remain unclear. Care is heavily physician-centric and focused on lab values and medication adjustments, meaning broader psychosocial needs (such as fatigue and return-to-work support) are largely neglected. Registered nurses reported lacking a formal, structured mandate or specialized training in thyroid care.

3. Work-Related Distress: This covers the emotional toll and restricted professionalism. Clinicians describe feeling helpless and experiencing moral injury when they cannot deliver the level of care patients need. Strained schedules limit professional judgment and raise fears of making medical mistakes.

Verbatim quotes are well-selected and highlight a real disconnect: while physicians are stressed by back-to-back, biochemically focused visits, nurses feel underutilized and underconfident. The narrative is convincing and directly answers the research question.

Interpretation and conclusions

The conclusions are realistic and supported by the data. The recommendation to introduce specialized "contact registered nurses" to coordinate care makes sense. It is a model that works well in diabetes, yet thyroid care remains stubbornly physician-centric.The authors rightly note that health economic evaluations of Swedish hyperthyroidism guidelines show team-based care with contact nurses to be cost-effective. They also point to a concrete solution: a 7.5-credit specialized course already being trialed at the University of Gothenburg to bridge the nursing competency gap.

References

The 35 references are relevant and up to date. They include Sweden's national hyperthyroidism guidelines, qualitative methods classics, and literature on physician burnout. There are no glaring omissions.

Abstract/summary/key messages

The abstract is accurate and matches the main text. It clearly summarizes the study's background, qualitative design, findings, and implications within the 359-word limit.

Reporting standards

The paper follows the Standards for Reporting Qualitative Research (SRQR) guidelines. The authors included a completed 22-item SRQR checklist in their appendix, which is excellent for transparency and research integrity.

**Reviewer**	**3**
**Name**	**Medenica, Sanja**
**Affiliation**	**University of Montenegro**
**Date**	**26-Jul-2026**
**COI**	**None**

Reviewer Comments

This is a well-written and clinically relevant qualitative study addressing an underexplored aspect of Graves' disease management. The manuscript provides valuable insights into the perspectives of healthcare professionals and highlights important organizational barriers that influence patient-centered care, interdisciplinary collaboration, and healthcare professional well-being. The findings have practical implications for improving the management of patients with Graves' disease and are highly relevant to clinicians and healthcare policymakers.

The methodology is appropriate, the qualitative analysis is rigorous, and the discussion is well supported by the existing literature. I have only a few minor suggestions that could further strengthen the manuscript:

Minor revisions are needed

Expand the discussion by briefly comparing your findings with healthcare delivery models for other chronic autoimmune diseases (e.g., diabetes or inflammatory rheumatic diseases), where multidisciplinary and nurse-led care has demonstrated improved patient outcomes.

Although the limitations are adequately described, it would be useful to include a brief comment on the potential influence of the Swedish healthcare system on the transferability of the findings to countries with different healthcare structures and resource availability.

Consider adding one or two sentences in the conclusion outlining future research directions, particularly intervention studies evaluating multidisciplinary care models, specialist thyroid nurses, or structured psychosocial support programs for patients with Graves' disease.

VERSION 1 - AUTHOR RESPONSE

Reviewer 1

Comment 1: *The participants' number was very small, and the findings obtained from the study were difficult to be generalised scientifically.*

Answer:
We thank the reviewer for this relevant comment.

We would like to clarify that the study was qualitative and aimed to explore participants' experiences in depth rather than to achieve statistical generalisability. In qualitative research, sample size is guided by the richness and relevance of the data in relation to the research question rather than by statistical power or the aim of achieving statistical representativeness. Recruitment and interviewing continued until the data were considered sufficiently rich to adequately address the research question and fulfil the study aim. We therefore consider the number of participants appropriate for the chosen qualitative methodology and do not regard the sample size itself as a limitation. While the sample is not intended to be statistically representative of a wider population, it captures relevant experiences and perspectives within the studied context. The findings are thus intended to provide an in-depth understanding of the phenomenon and may be transferable to similar contexts rather than statistically generalisable.

In response to the reviewer's comment, we have revised the manuscript to clarify the rationale for choosing a qualitative design (line 142-147, 149-151) and to more explicitly describe how trustworthiness and transferability were addressed (line 233-240). We have clarified that participants were recruited from different professional groups and hospital settings to obtain variation in experiences and perspectives (line 158-160,173-175).

Reviewer 2

We sincerely thank the reviewer for the thorough and positive assessment of our manuscript and for highlighting its originality, clinical relevance, and potential implications for clinicians, patients, healthcare managers, and policymakers. We particularly appreciate the reviewer's recognition of the appropriateness of the qualitative methodology and the value of including both physicians and registered nurses from different clinical settings.

Although the reviewer did not request specific revisions, several changes made in response to the other reviewers have further strengthened aspects highlighted in this assessment. Specifically, we have clarified the rationale for choosing a qualitative design (lines 142–147 and 149–151), the variation in professional and clinical settings represented in the participant sample (lines 158–160 and 173–175), and how trustworthiness and transferability were addressed (lines 233–240).

We have also expanded the Discussion to place our findings in a broader healthcare context by comparing them with multidisciplinary and nurse-led care models in rheumatoid arthritis and diabetes (lines 541-552). Furthermore, we have added a reflection on how the Swedish publicly funded healthcare system may influence the transferability of the findings to healthcare systems with different organisational structures and resource availability (lines 600-602, 606-612). Finally, the Conclusion has been expanded to highlight the need for future intervention studies evaluating multidisciplinary care models, specialist thyroid nurses, and structured psychosocial support for patients with GD (lines 635-641).

We thank the reviewer again for the encouraging and comprehensive evaluation of our work.

Reviewer 3

We sincerely thank the reviewer for the positive and constructive assessment of our manuscript and for highlighting its clinical relevance. We appreciate the suggestions that have helped us further strengthen the Discussion and Conclusion. We have addressed each comment as detailed below.

Comment 1: *Expand the discussion by briefly comparing your findings with healthcare delivery models for other chronic autoimmune diseases (e.g., diabetes or inflammatory rheumatic diseases), where multidisciplinary and nurse-led care has demonstrated improved patient outcomes.*

Answer: Thank you for this valuable suggestion. We agree that experiences from other chronic diseases provide a relevant perspective on our findings regarding the potential role of specialist nurses and multidisciplinary care in GD. We have therefore expanded the Discussion to include evidence from rheumatoid arthritis and diabetes, where multidisciplinary and nurse-led care models have been implemented. We have also added relevant references to systematic reviews in these areas. As these healthcare models have not been specifically evaluated in GD, we have been careful not to directly extrapolate their effects to GD but instead use them to illustrate the potential value of developing and evaluating similar approaches in GD care. The text is added to the discussion (lines 541-552).

Comment 2: *Although the limitations are adequately described, it would be useful to include a brief comment on the potential influence of the Swedish healthcare system on the transferability of the findings to countries with different healthcare structures and resource availability.*

Answer:

Thank you for this important comment. We agree that the healthcare context should be considered when assessing the transferability of our findings. We have therefore expanded the limitations section to acknowledge that the study was conducted within the Swedish publicly funded healthcare system. Although several of the challenges identified in our study, including time constraints, fragmented care pathways, and insufficient psychosocial support, have also been described internationally, differences in healthcare organisation, professional roles, access to specialist care, and resource availability may influence the applicability of our findings to other settings. This has now been clarified in the manuscript (lines 600-602, 606-612).

Comment 3. *Consider adding one or two sentences in the conclusion outlining future research directions, particularly intervention studies evaluating multidisciplinary care models, specialist thyroid nurses, or structured psychosocial support programs for patients with Graves' disease.*

Answer:

Thank you for this valuable suggestion. We agree that identifying future research directions strengthens the clinical implications of our findings. We have therefore added a statement to the Conclusion emphasizing the need for prospective intervention studies evaluating multidisciplinary care models, specialist thyroid nurses, and structured psychosocial support in GD (lines 635-641).

VERSION 2 - REVIEW

Reviewer 3

Name Medenica, Sanja

Affiliation University of Montenegro

Date 20-Sep-2026

COI

Congratulations I fifnd the manuscript to be accepted in this form.
